# Risk factors for TIVAD-related venous thrombosis in Chinese breast cancer patients undergoing chemotherapy: A single-center retrospective study

**DOI:** 10.1097/MD.0000000000048051

**Published:** 2026-03-20

**Authors:** Yin Li, Shuntao Wang, Xiangwang Zhao

**Affiliations:** a Department of Breast and Thyroid Surgery, Union Hospital, Tongji Medical College, Huazhong University of Science and Technology, Wuhan, P.R. China.

**Keywords:** breast carcinoma, fully implantable venous access device, risk determinants, thrombosis

## Abstract

Totally implantable venous access devices (TIVADs) are widely used in breast cancer patients receiving chemotherapy; however, catheter-related thrombosis can occur and may be asymptomatic. This study aimed to examine the prevalence and associated risk factors of TIVAD-related venous thrombosis in breast cancer patients undergoing chemotherapy. We conducted a retrospective analysis of 143 consecutive breast cancer patients who received TIVAD implantation at our hospital from March 2022 to February 2023. Routine color Doppler ultrasonography of catheter-associated veins was conducted following the conclusion of all chemotherapy cycles and prior to the removal of the TIVAD. Patients were classified into thrombosis and non-thrombosis cohorts. Variables relating to patients, treatments, and catheters were compared among groups. TIVAD-related venous thrombosis was detected in 37 of 143 patients (25.87%); all cases were asymptomatic and identified by routine ultrasound screening prior to TIVAD removal. The median TIVAD retention time was 148 days. A significant difference in TIVAD material was observed between groups (*P* < .05). No significant differences were found in age, body weight, tumor–node–metastasis (TNM) stage, puncture site, timing of chemotherapy, chemotherapy regimen category, catheter tip position, or TIVAD retention period (all *P* > .05). Asymptomatic TIVAD-associated venous thrombosis is prevalent among breast cancer patients undergoing chemotherapy. TIVAD material may be linked to thrombosis risk, although the duration of TIVAD retention was not substantially correlated with thrombosis in this population.

## 1. Introduction

Breast cancer is the most often diagnosed cancer in women globally, and systemic chemotherapy is a fundamental component of treatment at various stages of the disease.^[1-4]^ The frequent use of irritating or vesicant agents and supportive drugs necessitates dependable long-term venous access, rendering central venous access devices (CVADs) essential for numerous patients.^[5-7]^ Totally implantable venous access devices (TIVADs, implanted ports) and peripherally inserted central catheters (PICCs) are the 2 predominant CVADs utilized in chemotherapy for breast cancer.^[5,8,9]^ In contrast to PICCs, TIVADs are entirely implanted, facilitate continuous everyday activities, and are typically favored for extended, intermittent treatment regimens.^[10-12]^

Notwithstanding these benefits, catheter-related thrombosis (CRT) – comprising deep venous thrombosis and, less frequently, pulmonary embolism – persists as a clinically significant consequence.^[9,13,14]^ CRT may result in catheter malfunction, treatment disruption, supplementary anticoagulation, and heightened healthcare resource consumption.^[6,15,16]^ A significant percentage of CRT events are asymptomatic and may only be identified through image surveillance (e.g., Doppler ultrasonography) during follow-up or prior to device removal, thus leading to an underestimation of the actual burden in standard treatment.^[16,17]^

Patients with breast cancer may encounter a significant thrombotic burden due to a combination of cancer-related hypercoagulability, surgical interventions, and systemic therapy exposure.^[2,3,15]^ Endocrine therapy is extensively employed in breast cancer; population-based data suggest that the incidence of venous thromboembolism (VTE) increases shortly after the commencement of tamoxifen, but aromatase inhibitors have a significantly lower correlation.^[17-19]^ Moreover, analyses of thrombosis-related to cancer therapy have emphasized that tamoxifen may exacerbate the risk of VTE, especially in the presence of additional prothrombotic factors.^[15]^

Current evidence indicates that the risk of CRT is multifactorial, encompassing patient characteristics (age, body mass index, comorbidities), tumor- and treatment-related factors (such as disease stage and exposure to endocrine therapy), laboratory metrics (including lipid profile and coagulation indices), and catheter-related variables (puncture site, catheter tip position, and device attributes).^[14,17,18,20]^ Furthermore, the selection of devices may affect thrombotic risk: recent meta-analyses indicated elevated overall complication rates associated with PICCs in comparison to implanted ports, with thrombosis being a significant factor.^[8,12]^ Nevertheless, data about TIVAD-associated venous thrombosis in Chinese breast cancer patients is scarce, and the factors contributing to asymptomatic thrombosis detected during routine imaging before port removal are little defined. Consequently, we performed a single-center retrospective analysis to assess the incidence and risk factors of TIVAD-related venous thrombosis in breast cancer patients receiving chemotherapy, with the objective of guiding personalized risk stratification and device management methods.

## 2. Materials and methods

### 2.1. Design and setting

We used data from the the Haitai Medical information system clinically used by Union Hospital Affiliated to Huazhong University of Science and Technology in Wuhan, China. Data of patients with breast cancer who were admitted to the breast department and underwent chemotherapy from March 2022 to February 2023 were retrospectively collected by the specialized research assistants after obtaining the informed consent from the patients. The study protocol was censored and approved by the Institutional Ethics Committee of Union Hospital, Tongji Medical College, Huazhong University of Science and Technology. Patient consent was achieved by informed consent forms. The study was conducted in accordance with the Declaration of Helsinki. The primary objective of this study was to identify patient-, treatment-, and catheter-related factors associated with TIVAD-related venous thrombosis in breast cancer patients undergoing chemotherapy. The secondary objectives were: to determine the incidence of TIVAD-related venous thrombosis detected by routine color Doppler ultrasonography at the completion of chemotherapy prior to TIVAD removal and to describe thrombosis characteristics, including the time from TIVAD implantation to thrombosis detection. This study was conducted in accordance with the Declaration of Helsinki and was approved by the Ethics Committee of Union Hospital, Tongji Medical College, Huazhong University of Science and Technology. Written informed consent was obtained from all participants. Sample size justification: the sample size was determined based on the estimated incidence of TIVAD-related venous thrombosis in breast cancer patients undergoing chemotherapy (25% according to previous retrospective studies.^[21]^ Using a 2-sided test with α = 0.05 and β = 0.2 (statistical power = 80%), the minimum required sample size was calculated as 120 cases. We consecutively included 143 eligible patients to ensure sufficient statistical power for detecting potential associations between candidate risk factors and thrombosis.

### 2.2. Study population and materials

During March 2022 to February 2023, consecutive breast cancer patients undergoing first-time TIVAD implantation for chemotherapy were retrospectively screened, and 143 eligible patients were included after excluding those with prior VTE, previous breast cancer/chemotherapy, prior TIVAD (or other CVAD) history, clinically significant coagulation disorders, or missing pre-removal Doppler ultrasonography follow-up. The inclusion criteria were: age ≥ 18 years; receipt of chemotherapy with a TIVAD in the study period; and first-time TIVAD implantation for the index chemotherapy course. The exclusion criteria were: prior history of VTE, including deep venous thrombosis or pulmonary embolism; previous history of breast cancer and/or prior chemotherapy before the index TIVAD implantation; any prior history of TIVAD implantation or other CVADs; known hematologic diseases or clinically significant coagulation disorders; and missing follow-up color Doppler ultrasonography assessment of catheter-associated veins prior to TIVAD removal.

Data collected from patients include: general information of patients (age, weight, etc); the disease status of the patients (pathological type of breast cancer, chemotherapy timing, chemotherapy regimen, disease stage); and the patient’s TIVAD catheter placement (material, location and depth of implanted TIVAD catheter). The TIVAD retention period (retention time) was defined as the duration from TIVAD implantation to TIVAD removal (days). Patients were categorized into the thrombosis group and the non-thrombosis group according to routine color Doppler ultrasonography of catheter-associated veins performed after completion of chemotherapy and prior to TIVAD removal. The thrombosis group was defined as patients with ultrasound-confirmed catheter-related venous thrombosis, whereas the non-thrombosis group was defined as patients without evidence of thrombosis on ultrasonography. All the variables reported in our study were completely measured in all participants.

#### 2.2.1. The procedure of TIVAD implantations and maintenance

According to the standard procedure of our center, the internal jugular vein was considered a priority without abnormal conditions (internal jugular vein thrombosis, tumor infiltration, or infection, puncture failure). Two brands of ports, Bard (Bard Limited, USA) and Intra (Intra special catheters GmbH, Germany) were chosen according to the surgeon’s preference. Patients were placed in a supine position on the operation table. The pectoral region of the planned implantation side, as well as the collar region, was scrubbed with betadine and a maximum sterile barrier precaution was provided. In order to prevent clot formation and catheter blockage, TIVAD was flushed with 20 mL saline before implantation. Local anesthesia was administrated subcutaneously, restricted to the port implantation and the venous puncture area. After local infiltration anesthesia by the surgeon, the left internal jugular vein or right intravenous vein was selected at an average position of 2 to 3 cm above the clavicle with an 18-gauge needle under the guidance of continuous ultrasonic guidance for internal jugular vein puncture and catheter placement. After the implantation, the port seat of TIVAD was buried in the ipsilateral subclavian subcutaneous tissue space, and the correct positioning of the tip of the catheter was confirmed by X-ray to be localized in the vena cava superior. Experienced nurses flushed the TIVAD every 21 days with 10 mL of a heparin-sodium solution 100 IE/mL or at the end of each cycle of chemo-therapy infusions to maintain patency. No prophylaxis anticoagulant was given during chemotherapy, no attempt was made to detect occult thrombosis during chemotherapy and no symptomatic thrombosis occurred. Following the chemotherapy cycle, the patients underwent a follow-up examination using color ultrasonography of the neck vasculature to assess the catheter-associated vein before the TIVAD being removed to detect CRT existence. The asymptomatic CRT detected prior to TIVAD removal was treated according to each doctor’s clinical practice.

The possible factors that affect TIVAD-related thrombosis were extracted and analyzed in this study, including age, body mass index, TNM stage, puncture site (left/right internal jugular vein), timing of chemotherapy (before/after surgery), chemotherapy regimen, catheter tip position, catheter retention time, and TIVAD material (Bard or Intra). Hormone receptor status (estrogen receptor [ER] and progesterone receptor [PR]) and thrombosis-related laboratory parameters (LDL, HDL, total cholesterol, triglycerides, prothrombin time, activated partial thromboplastin time, and D-dimer) were not routinely recorded/available in our retrospective dataset; therefore, these variables could not be included in the present analysis. Consistency of assessment methods across groups: all patients in the thrombosis and non-thrombosis groups underwent the same standardized color Doppler ultrasonography protocol (using the same ultrasound device and detection criteria) for assessing catheter-associated veins prior to TIVAD removal. Data extraction was performed by specialized research assistants following a unified data collection form, ensuring comparability of measurement methods between the 2 groups. Based on the available variables, patients with TIVAD-associated thrombosis were categorized into predefined groups and venous thrombosis rates were analyzed accordingly.

### 2.3. Statistical analysis

Loss to follow-up: due to the retrospective design and routine clinical follow-up procedures at our institution, all included patients completed the planned follow-up (i.e., color Doppler ultrasonography prior to TIVAD removal) without loss to follow-up. Thus, no specific statistical methods for handling loss to follow-up were required. The mean ± standard deviation or median (interquartile range [IQR]) were used to express continuous data points. Categorical data are expressed as number (percentage [%]). The Shapiro–Wilk test was used to check the normality of the data. Normally distributed data were compared between the groups using the 2 sample independent *t* tests. Categorical data were compared using the Chi-square or Fisher’s exact test. Ordinal data and non-normally distributed continuous data were calculated using the Mann–Whitney *U*-test to test differences between groups for their statistical significance. There were no missing values for the variables examined in the statistical analysis. Analyses were conducted using IBM SPSS Statistics (Version 20, IBM Corporation, Armonk). All tests were 2-sided, and a *P*-value of <.05 was considered statistically significant.

## 3. Results

A total of 143 consecutive patients (all female) with pathologically confirmed breast cancer who underwent first-time TIVAD implantation for chemotherapy were included in this retrospective analysis. Due to the retrospective nature of the study and the long interval since data collection, detailed records of the total number of potentially eligible patients screened were not available. However, all included patients strictly met the predefined inclusion and exclusion criteria (listed in the Methods section), and no patients were excluded after enrollment due to incomplete data or loss to follow-up. Among them, 25.87% (37/143) suffered CRT during the chemotherapy period, all of which were asymptomatic and detected by routine ultrasound evaluation before TIVAD removal when chemotherapy was complete. All 37 cases of thrombosis were asymptomatic at diagnosis and were identified through routine Doppler ultrasonography screening performed at the completion of chemotherapy prior to TIVAD removal. There was no thrombosis detected in the internal jugular vein, and all the CRT was attached to the catheter surface (Table 1). These 37 patients were assigned to the thrombosis group, and the other 105 patients with no detected CRT were assigned to the no thrombosis group. All variables analyzed in this study (age, body weight, TNM stage, puncture site, timing of chemotherapy, chemotherapy regimen, catheter tip position, TIVAD material, and TIVAD retention time) were completely recorded in the medical information system, with no missing values among the 143 included patients.

**Table 1:** Association between risk factors and TIVAD-associated thrombosis.

	No thrombosis group (n = 105)	Thrombosis group (n = 37)	χ^2^value	*P*-value
Age, years old				
≥60	19 (18.1%)	5 (13.5%)	0.409	.523
<60	86 (81.9%)	32 (86.5%)		
BMI	59.28 ± 8.076	60.19 ± 9.854		.578
Stage of TNM			0.006	.937
I–II	97 (92.4%)	35 (94.6%)		
III–IV	8 (7.6%)	2 (5.4%)		
Puncture site (Internal jugular vein)			0.146	.702
Left	23 (21.9%)	7 (18.9%)		
Right	82 (78.1%)	30 (81.1%)		
Timing of chemotherapy			1.778	.182
Before surgery	35 (33.3%)	8 (21.6%)		
After surgery	70 (66.7%)	29 (78.4%)		
Chemotherapy drugs			2.67	.263
AC-T	12 (11.4%)	7 (19.0%)		
EC-T	38 (36.2%)	16 (43.2%)		
Others	55 (52.4%)	14 (37.8%)		
Retention time of catheter, d	148 (135,168)	148 (137,153)		.345
Catheter tip position			3.675	.452
T5	1 (1%)	0		
T6	15 (14.3%)	8 (21.6%)		
T7	63 (60%)	24 (64.9%)		
T8	25 (23.8%)	5 (13.5%)		
T9	1 (1%)	0		
Material of catheter			4.343	.037*
Bard	53 (50.5%)	26 (70.3%)		
Intra	52 (49.5%)	11 (29.7%)		

Data are number (percentage), mean (standard deviation) or median (interquartile range). TIVAD retention period/time indicates the duration from TIVAD implantation to device removal (days).

AC-T = doxorubicin and cyclophosphamide followed by docetaxel, BMI = body mass index, EC-T = epirubicin and cyclophosphamide followed by docetaxel, TIVAD = totally implantable venous access device, TNM = tumor node metastasis.

* *P* < .05, indicating a statistically significant difference.

In the thrombosis group, the age distribution of patients was 86.5% (32/37) aged <60 years and 13.5% (5/37) aged ≥60 years. In the no thrombosis group, 81% (86/105) of patients were aged <60 years and 18.1% (19/105) were aged ≥60 years. As shown in Table 1, there were no significant differences between the thrombosis and non-thrombosis groups in age, body weight, TNM stage, puncture site, timing of chemotherapy, chemotherapy regimen category, or catheter tip position (all *P* > .05). The median TIVAD retention period between the 2 groups was 148 days. Here, “TIVAD retention period” refers to the device dwelling duration from implantation to removal after completion of chemotherapy. The difference was statistically significant in the material of the TIVAD between the 2 groups (*P* < .05). In the thrombosis group, 70.3% of patients (26/37) used TIVADs made by Bard (Bard Limited, USA), and 29.7% of patients (11/37) used TIVADs made by Intra (Intra Special Catheters GmbH, Germany). Among the patients in the no thrombosis group, 50.5% (53/105) used the TIVAD made by Bard (Bard Limited, USA), and 49.5% (52/105) used the TIVAD made by Intra (Intra special catheters GmbH, Germany). Following 4 weeks of anticoagulant therapy, all 37 patients made a full recovery, and the CRT disappeared completely. The TIVAD was successfully withdrawn at the end of chemotherapy without aggravating thrombosis, pulmonary embolism or hemorrhage.

## 4. Discussion

This single-center retrospective analysis of Chinese breast cancer patients undergoing chemotherapy with TIVADs aimed to investigate the incidence and risk factors of TIVAD-related venous thrombosis. Consistent with our primary objective, we found that the incidence of asymptomatic TIVAD-related venous thrombosis was 25.87% (37/143), as detected by routine color Doppler ultrasonography prior to TIVAD removal. Regarding our secondary objectives, we identified TIVAD material as a potential risk factor (Bard vs Intra, *P* < .05), while age, body weight, TNM stage, puncture site, timing of chemotherapy, chemotherapy regimen, catheter tip position, and TIVAD retention time were not significantly associated with thrombosis risk. Additionally, the median TIVAD retention time was 148 days, which did not show a significant correlation with thrombosis.

Prior breast cancer research has indicated significant fluctuation in the incidence of CRT, primarily contingent upon whether occurrences are detected solely by clinical presentation or via routine imaging surveillance.^[22-24]^ In breast cancer cohorts utilizing ultrasound screening before port removal, asymptomatic thrombosis constitutes a significant percentage of CRT events and results in elevated overall CRT rates compared to symptom-driven identification alone.^[20,21,25]^ Consequently, the incidence noted in our study may be partially attributed to the systematic ultrasonography assessment conducted prior to device removal, which enhances the identification of clinically silent thrombosis.

The selection of venous access device may affect thrombotic risk in breast cancer.^[2,3,26]^ Meta-analyses examining breast cancer cohorts have contrasted implantable ports with PICCs, revealing variations in complication profiles, particularly with thrombosis as a significant factor, despite considerable variability across the trials analyzed.^[12,27,28]^ As all participants in our study were administered TIVADs, device-type confounding was mitigated, facilitating a more concentrated assessment of patient- and device-related variables within the implanted port context.

A relationship was also identified between thrombosis and the manufacturer/material of TIVAD.^[14,15,21]^ Causality cannot be deduced from this retrospective analysis; nonetheless, the features of catheter surfaces and materials may influence thrombogenicity via endothelial irritation, fibrin sheath creation, platelet/fibrin deposition, and local flow disruption.^[28-30]^ These findings indicate that device-related attributes should be factored into clinical decision-making and require validation in prospective trials employing standardized implantation and follow-up protocols.

We did not observe statistically significant variations in catheter tip positioning or retention duration. This may indicate a pretty uniform implantation practice and minimal fluctuation in these parameters within a single institution. Furthermore, chemotherapy regimens were assessed as general categories; hence, the restricted granularity of exposure and sample size may have diminished the capacity to identify regimen-specific correlations, which cannot be ruled out.^[18,23,31,32]^

Multiple constraints must be recognized. First, this was a single-center retrospective study with a relatively small sample size, which may limit the generalizability of the results and increase the risk of selection bias. Specifically, the patient population was limited to those treated at a single tertiary hospital in Central China, and the association between TIVAD material and thrombosis may be overestimated due to the lack of external validation. Second, due to the retrospective design and long data collection interval, detailed records of the total number of screened patients and individual exclusion reasons (beyond the predefined criteria) were unavailable, which may introduce potential selection bias. Third, numerous clinically relevant parameters (e.g., ER/PR status, D-dimer levels) were not uniformly documented, limiting the comprehensiveness of risk factor analysis.^[21,33]^ Fourth, the precise onset time of thrombosis could not be determined, as ultrasound was only performed once prior to TIVAD removal. The findings of this study may be generalizable to female breast cancer patients who undergo first-time TIVAD implantation for chemotherapy in similar tertiary hospitals in Central China, as the patient characteristics, TIVAD implantation procedures, and chemotherapy regimens used in our institution are representative of standard clinical practice in this region. However, caution should be exercised when extending these results to other populations (e.g., patients in primary hospitals, those with comorbidities not included in the analysis, or patients using different TIVAD brands), as these factors may affect the risk of TIVAD-related thrombosis. Multicenter retrospective or prospective studies with larger sample sizes are needed to validate our findings.

## 5. Conclusion

This single-center retrospective cohort study of Chinese breast cancer patients receiving chemotherapy with TIVADs identified asymptomatic catheter-related venous thrombosis in 25.87% of patients, as determined by Doppler ultrasonography conducted before elective device removal. The TIVAD manufacturer/material was linked to thrombosis, while age, body weight, TNM stage, puncture side, timing of chemotherapy, chemotherapy regimen category, catheter tip position, and TIVAD retention period showed no significant differences between groups. The findings indicate that clinically silent CRT is relatively common and suggest that characteristics related to the device may influence the risk of thrombosis, potentially guiding device selection and monitoring strategies in clinical practice.

This study is constrained by its retrospective single-center design, limited sample size, and incomplete data on several clinically relevant variables, such as ER/PR status and thrombosis-related laboratory biomarkers. Additionally, the precise onset time of thrombosis could not be established due to the predetermined timing of ultrasound assessment. Future multicenter studies utilizing standardized longitudinal imaging alongside comprehensive clinical and laboratory data are necessary to validate our findings, elucidate mechanisms, and establish effective risk stratification and prevention strategies for TIVAD-related thrombosis in breast cancer patients.

## Acknowledgments

The authors thank all participants and the clinical staff involved in data collection. This study received no external funding. The authors declare that they have no conflicts of interest.

## Author contributions

**Conceptualization:** Shuntao Wang, Xiangwang Zhao.

**Data curation:** Yin Li, Shuntao Wang.

**Formal analysis:** Yin Li, Shuntao Wang, Xiangwang Zhao.

**Investigation:** Yin Li.

**Project administration:** Shuntao Wang.

**Resources:** Shuntao Wang, Xiangwang Zhao.

**Software:** Yin Li.

**Validation:** Yin Li.

**Visualization:** Yin Li.

**Writing – original draft:** Yin Li.

**Writing – review & editing:** Shuntao Wang, Xiangwang Zhao.
